# Intragraft Selection of the T Cell Receptor Repertoire by Class I MHC Sequences in Tolerant Recipients

**DOI:** 10.1371/journal.pone.0006076

**Published:** 2009-06-29

**Authors:** Dahai Liu, Xiu-Da Shen, Yuan Zhai, Wengsi Lam, Jingying Liao, Ronald W. Busuttil, Rafik M. Ghobrial

**Affiliations:** 1 The Dumont-UCLA Transplant Center, Division of Liver and Pancreas Transplantation, Department of Surgery, David Geffen School of Medicine, Los Angeles, California, United States of America; 2 Department of Surgery, The Methodist Hospital, Houston, Texas, United States of America; New York University School of Medicine, United States of America

## Abstract

**Background:**

Allograft tolerance of ACI (RT1^a^) recipients to WF (RT1^u^) hearts can be induced by allochimeric class I MHC molecules containing donor-type (RT1A^u^) immunogenic epitopes displayed on recipient-type (RT1A^a^) sequences. Here, we sought the mechanisms by which allochimeric sequences may affect responding T cells through T cell receptor (TCA) repertoire restriction.

**Methodology/Principal Findings:**

The soluble [α_1h_^u^]-RT1.A^a^ allochimeric molecule was delivered into ACI recipients of WF hearts in the presence of sub-therapeutic dose of cyclosporine (CsA). The TCR Vβ spectrotyping of the splenocytes and cardiac allografts showed that the Vβ gene families were differentially expressed within the TCR repertoire in allochimeric- or high-dose CsA-treated tolerant recipients at day +5 and +7 of post-transplantation. However, at day 30 of post-transplantation the allochimeric molecule-treated rats showed the restriction of TCR repertoire with altered dominant size peaks representing preferential clonal expansion of Vβ7, Vβ11, Vβ13, Vβ 14, and Vβ15 genes. Moreover, we found a positive correlation between the alteration of Vβ profile, restriction of TCR repertoire, and the establishment of allograft tolerance.

**Conclusions:**

Our findings indicate that presentation of allochimeric MHC class I sequences that partially mimic donor and recipient epitopes may induce unique tolerant state by selecting alloresponsive Vβ genes.

## Introduction

The possible mechanisms underlying the development of transplantation tolerance include clonal deletion of alloreactive cells, clonal anergy, cell-mediated suppression, and “infectious” tolerance [1]. Studies on the mechanisms responsible for the establishment of transplantation tolerance point to the immune regulation as the major determinant. It was shown previously that the CD4+ T lymphocytes from tolerate hosts inhibit rejection of donor-specific allografts after adoptive transfer into test rat recipients [2] and the examination of tolerant cardiac allografts in both early and late post-transplantation stages indicated that in contrast to the rejecting grafts they were extensively infiltrated by the T cells [3], [4]. Following allotransplantation, the T cells can recognize graft MHC antigens in two different ways: via “direct” recognition of antigens on donor cells and via “indirect” recognition of processed donor antigens presented on recipients APCs in the form of peptides combined with MHC molecules [5]. Numerous studies on rodents and humans demonstrate that the indirect recognition of MHC plays a major role in both acute and chronic allograft rejection [6]–[8].

In response to alloreaction, T cells react to allogeneic MHC molecules by displaying a dominant determinant on donor MHC antigens and are restricted by self-MHC elements [9]–[11]. It was shown previously that the alteration of critical amino acid residues in such immunodominant determinants inhibited T cell proliferative responses, induced T cell anergy and altered host immune responses toward native antigens [12]–[15]. Furthermore, analysis of T cell responses to nominal protein or transplantation antigens has shown that constraints in antigen processing and presentation usually limit the T cell responses to one or two dominant determinants [3], and that, in humans, T cells responding to dominant allopeptides had limited TCR-Vβ gene usage [10], [11]. Thus, indirect allorecognition by T cell possibly regulates specific immunointervention [16]–[19] through manipulation of such dominant immunogenic epitopes.

Previously we have mapped an immunogenic determinant for rat class I RT1**^u^**, RT1**^l^** and RT1**^a^** alloantigens to amino acid residues 58–80 on α_1_-helical polymorphic region [20], [21]. MHC alloantigens are therefore similar to multideterminant protein antigens that bear localized immunodominant amino acid sequences that trigger immune responses toward the whole protein molecule [22]–[25]. Therefore, monitoring the profile of alteration of TCR repertoire will provide a proof of indirect recognition of MHC molecules through TCR restriction.

Alterations in the length distribution of complementary-determining region 3 (CDR3)^3^ of TCR Vβ have been observed *in vitro* and *in vivo* during allorecognition [5]. Indirect [α_1h_^u^]-RT1.A^a^ allochimeric recognition may induce functionally unique regulatory T cells with distinctive TCR allospecificities. To test this hypothesis, we have performed a comprehensive analysis of TCR Vβ gene usage in parallel with CDR3 spectrotyping of T cells. The CDR3 length distribution (CDR3-LD) measurement of the different Vβ genes demonstrated the antigenic diversity recognized by a T cell population. Our experiments provide direct evidence of the ability of indirectly presented allochimeric sequences to select the TCR repertoire and thus provide a powerful tool for manipulating the immune response through the sequences of the indirectly presented antigen. The characteristics of the TCR Vβ expression pattern may potentially be used as a novel marker to identify operational regulatory T cells in the recipient of organ allograft.

## Results

### Induction of Tolerance by [α_1h_^u^]-RT1.A^a^ to WF (RT1.A^u^) Allograft in ACI (RT1.A^a^) Recipient

We had constructed an immunogenic [α_1h_^u^]-RT1.A^a^ molecule by altering hypervariable α_1_-helical region (a.a. 51–90) of ACI (RT1.A^a^) to WF (RT1.A^u^) sequences [20], [26]. The resulted [α_1h_^u^]-RT1.A^a^ molecule bears both RT1.A^u^ and RT1.A^a^ α_1_-helical epitopes. We have previously demonstrated [20], [26] that peri-transplant portal venous (p.v.) administration of [α_1h_^u^]-RT1.A^a^ allochimeric molecules induced tolerance to WF (RT1^u^) heart allografts in ACI (RT1^a^) recipients, when administered in conjunction with sub-therapeutic dose of CsA [31]. This indicates that peri-transplant administration of syngeneic class I sequences flanking allogeneic α_1_-helical immunogenic epitopes, in combination with a brief sub-therapeutic dose of CsA, is able to induce donor-specific transplantation tolerance.

### TCR Responses in Long-Term Tolerant Allochimeric Conditioned Recipients

**Figure 1:**
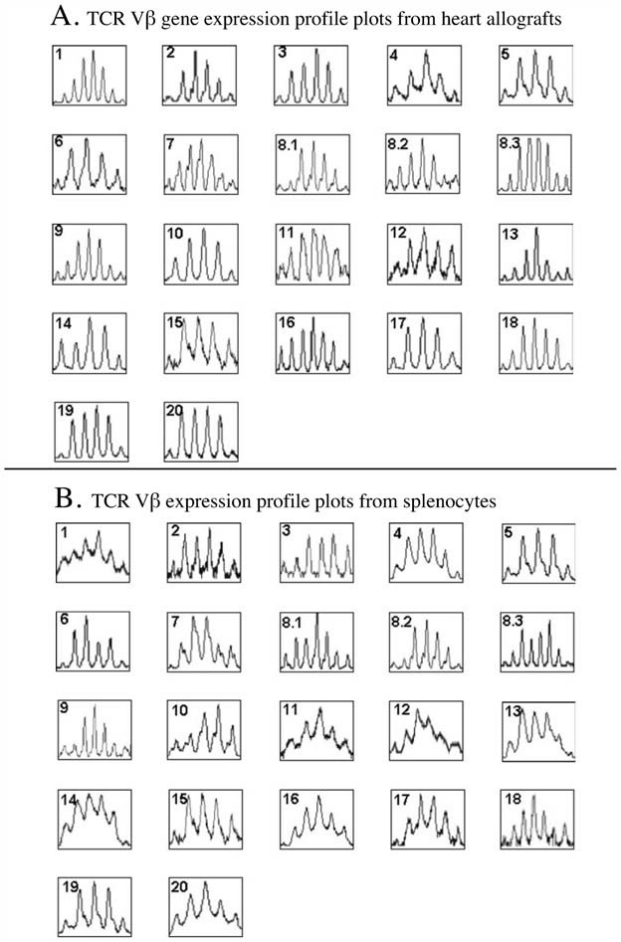
RT-PCR-assisted detection of expression profile of 22 known rat TCR Vβ genes in CsA-induced long-term tolerant ACI recipients bearing WF heart allografts (survival day>100). (A) TCR Vβ gene expression profile plots from heart allografts. A fluorochrome-labeled primer derived from β chain constant region was used for run-off reactions for each Vβ-specific RT-PCR product, and the fluorescent run-off products were analyzed in 3700 DNA Analyzer, as described in *Materials and Methods*. Panel numbers correspond to the numbers of individual Vβ genes. (B) TCR Vβ expression profile plots from splenocytes.

CDR3 spectrotyping shows three to seven visible bands of different size for each Vβ gene product, which intensities follow Gaussian distribution. Twenty two samples of TCR Vβ profile plots (derived from DNA Analyzer and Genotyper 3.7 software) from CsA-treated long-term tolerant ACI hosts bearing allochimeric [α_1h_^u^]-RT1.A^a^ class I allografts are shown in Fig. 1A. The TCR Vβ profile plots from the allografts of CsA-induced long- term tolerant recipients were similar to the profile plots from the recipients' spleen cells (Fig. 1B). In several profile plots (e.g., Vβ3, 8.1, 13, 14, and 19) we observed minor alterations in pick distribution patterns between allograft and spleen cells. Because these alterations were inconsistent between experiments we believe that they represent data output “noise” inherent to the applied method of analysis. In summary, it seems that a broad immunosuppressive agent such as CsA is unable to induce a clonal restriction of TCR.

**Figure 2:**
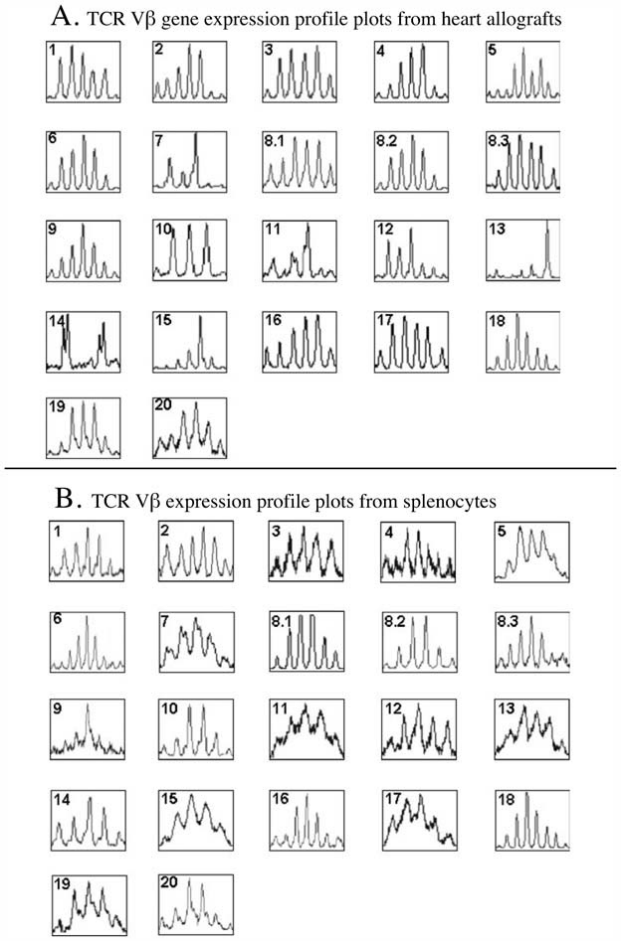
RT-PCR-assisted detection of expression profile of 22 known rat TCR Vβ genes in allochimeric protein [α_1h_^u^]-RT1.A^a^ class I molecule -induced long-term tolerant ACI recipients bearing WF heart allografts (survival day>100). (A) TCR Vβ gene expression profile plots from heart allografts. (B) TCR Vβ gene expression profile plots from splenocytes.

In all (n = 12) examined long-term (120 days) tolerant hosts induced by allochimeric protein [α_1h_^u^]-RT1.A^a^, all twenty two TCR Vβ genes exhibited constant restriction with altered dominant size peaks in Vβ7, 11, 13, 14, 15 (Fig. 2A). We also observed the alteration in the regular Gaussian distribution of 7–11 peaks in the following Vβs: 1, 2, 3, 4, 5, 8.1, 10, 12, and 16.

To confirm that restrictions of TCR repertoire (i.e. the oligoclonal expansion of T cells) were unique to the graft site rather than being a general feature of T cells of tolerant recipients, we also performed spectrotyping of the host splenocytes. Figure 2B shows that, indeed, these unique dominant peaks were detected exclusively in heart allografts, and were absent in splenocytes from the same tolerant host. Thus, all restrictions of TCR repertoire observed in our studies were allograft-specific.

### Frequency of Oligoclonality of T Cells in Tolerated Grafts

**Figure 3:**
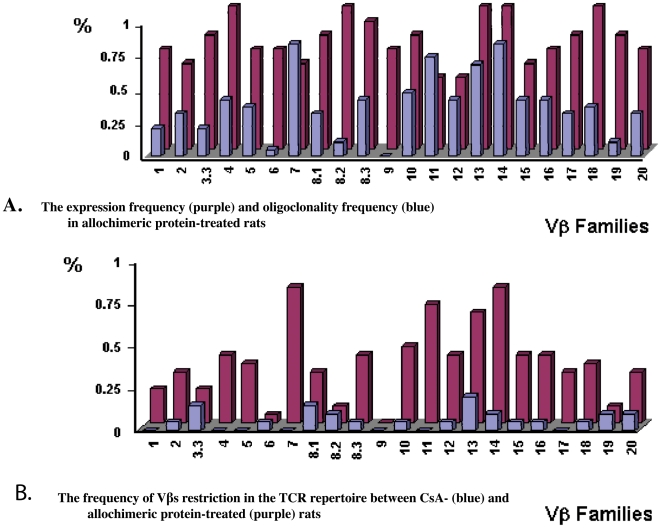
The frequency of oligoclonality and of Vbs expression in TCR repertoire in long-term (>100 days) tolerant CsA and allochimeric protein-treated, ACI recipients of WF cardiac allografts. The frequency of expression and oligoclonality for each Vb gene was calculated as a percentage of the total frequency of detection of each Vb in all (positive and negative) PCR amplification experiments. (b) There is significant difference between the frequency of Vbs restriction in the TCR repertoire between CsA- (blue) and allochimeric protein-treated (purple) rats. (a) There was no correlation between expression frequency (purple) and oligoclonality frequency (blue) in allochimeric protein-treated rats. Vb7, 11, 13, 14, 15 gene products show 1-3 dominant peaks, indicating TCR restriction in the T cell repertoire in allochimeric protein-induced tolerant rats.

The algorithm for calculating the percentage of alterations of expression profile gene and percentage of expression frequency for each Vβ gene were performed as described in the legend to Fig. 3A. We found that the expression frequencies of individual Vβ genes varied in a series of twelve grafts from allochimeric protein-treated rats (Fig. 3A). On the other hand, the Pearson correlation analysis indicated that there was no significant difference between the expression frequency of various Vβs in CsA-treated and allochimeric-protein treated rats (Correlation coefficient = −0.00938; *P* = 0.9669).

Although all twenty two Vβ gene products were readily detectable in each individual graft from both CsA and allochimeric protein-treated tolerant rats, the overall Vβ usage was restricted only in allochimeric protein-treated rats. As summarized in Figure 3B, the restriction frequencies of individual Vβ genes varied in grafts from allochimeric protein-treated tolerant rats: the Vβ4, 8.3, 10, 12, 16 were slightly restricted in most of the transplants and Vβ7, 11, 13, 14, 15 displayed dominant restricted bands, while Vβ1, 3.3, 8.2, 9, 19 were expressed much less frequently (Fig. 3A and 3B). Interestingly, there was no correlation between the frequency of expression of particular Vβ genes and their restriction level (Fig. 3A). This indicates that only selective Vβ gene families underwent significant clonal expansion and that the restriction of Vβ genes was not related to their expression level.

Interestingly, CDR3 spectrotyping of Vβ gene products showed remarkable differences between allochimeric-protein- and CsA-treated rats. Two-sample *t-test* analysis of the differences of the detection frequency of Vβ restricted in total TCR repertoire between CsA and allochimeric protein-induced tolerant rats showed 6% restriction frequency in CsA-treated tolerant allograft versus 36% in allochimeric protein -treated allograft (Fig. 3B). This difference was statistically significant (t = 6.2; *P<0.0001*).

### Dynamic Alteration of T Cell Receptor Repertoire in CsA and Allochimeric Conditioned Long-Term Tolerant Hosts

**Figure 4:**
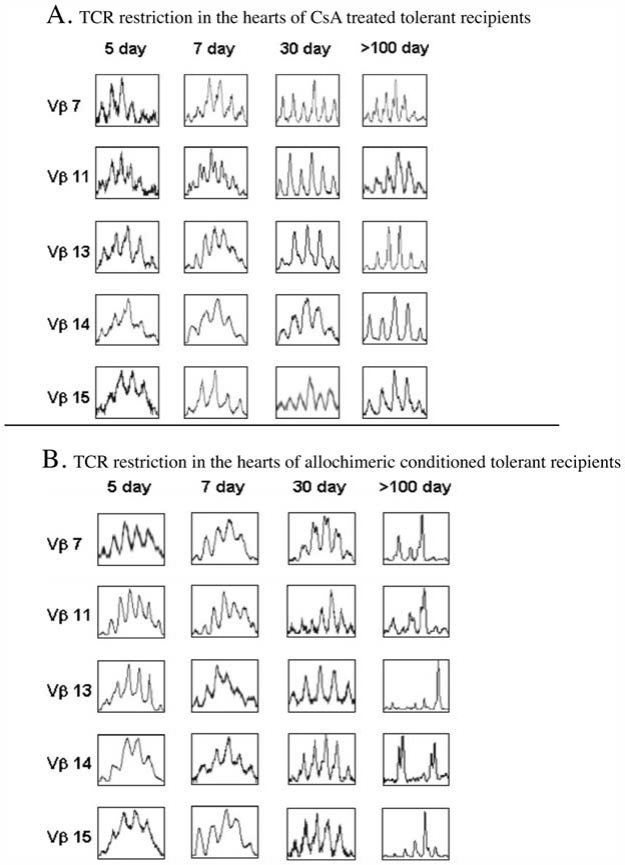
The dynamic alteration of T cell receptor repertoire in CsA (A) and allochimeric conditioned (B) long-term tolerant hosts. RNAs from heart allografts were harvested at different time points after transplantation followed by TCR detection. There were >7 samples for each time-point.(B) (A) There was no TCR restriction in the hearts of CsA treated tolerant recipients throughout the whole period of long-term tolerance (>day 120). (B) There was no TCR restriction in the hearts of allochimeric protein-treated tolerant recipients until 30 days post-transplantation. However, the Vβ7, 11, 13, 14 and15 were highly restricted and occurred as the dominant peaks 84 days after transplantation. This restriction was maintained throughout the whole period of long-term tolerance (>day 120),

To analyze the restriction pattern of TCR repertoire, the RNA was harvested at the different post-transplantation time points, at day 5, 7, 30, and >100 from CsA- and allochimeric protein-treated tolerant rats and then subjected to CDR3 spectrotyping (Fig. 4). The restriction of TCR repertoire in allochimeric protein conditioned tolerant recipients did not occur before 30 days of post-transplantation, and dominant CDR3 bands in Vβ7, 11, 13, 14, 15 gene products were found more frequently at 100 days post-transplantation (Fig. 4B). In contrast, nearly all Vβ gene products were unrestricted throughout the whole post-transplantation period in CsA induced long-term tolerant hosts (Fig. 4A).

## Discussion

Our previous data suggested that tolerance might be induced by functional presentation of donor immunogenic epitopes via self-sequences [20], [26]. Since processing of allochimeric molecules may alter the repertoire of alloreactive T cells, we applied CDR3 spectrotyping of TCR repertoire to analyze the clonal expansion of T cells in allografted hearts of tolerant recipients. Spectrotyping has been successfully used to analyze T cell responses to some well-characterized peptide antigens *in vivo* [31]–[33]. In current studies, we first determined whether there is any preferential accumulation of T cells with limited Vβ expression at the graft site. As the CDR3 spectrotyping can detect T cell clones against a background of polyclonal cells at a frequency of at least 1 in 1000 [34]–[36], we then used this novel technique to identify clonally expanded T cells by comparing CDR3 patterns in CsA and allochimeric protein-treated rats. We found that most of Vβ genes were expressed at the similar levels in long-term tolerant rats induced by CsA and allochimeric treatment and clonal expansion occurred only in a limited number of Vβ genes and was consistently observed in Vβ7, 11, 13, 14, 15. In contrast, no restricted clonal expansion was found in either spleencytes or CsA treated tolerant. The restricted clonal expansion observed in our study may indicate the presence of suppressed alloreactive T cells or preferential activation of putative regulatory T cells. Based on our previous findings, we argue that these cells are the regulatory cells [30], [38], [40]. Functional relevance of the oligoclonal T cell expansion can be addressed by using Vβ-specific mAb to selectively target specific Vβ gene products [41]. However, at present, this method is limited because only few anti-Vβ antibodies are available. An alternative method to test the functional relevance of T cell oligoclonality is the newly developed technique of TCR-specific DNA vaccination that inactivates targeted T cell clone(s) [42]. Our current study is the first to document a unique pattern of CDR3 spectrotyping in the tolerance pathway induced by allochimeric protein. Thus, our experimental design will allow us to further address the mechanism through which the indirectly presented allochimeric sequence could shape the responding TCR repertoire, which has many important implications for tolerance induction and inhibition of chronic rejection. Identification of the specific T cells involved in these processes may also provide an important tool to design intervening reagents in clinical treatment.

The mechanism of the recognition of allochimeric MHC molecules and T cells remains unclear. It was hypothesized that T cells selected by self-MHC molecules in the thymus could proliferate and mutate their receptors to allow them preferentially to recognize “altered-self” MHC molecules [39], [43], [44]. A restricted oligoclonal rather than a single clonal expansion of the Vβ TCR repertoire observed in our study indicates a pattern of hierarchical immunodominance or a cross-reactive response to cryptic self epitopes when regulatory cells are generated in response to self-peptide antigens. This issue can be addressed by dissecting the TCR repertoire using a second allochimeric molecule which shares similar self-RT1.A^a^ sequences to allochimeric molecule [α_1h_ **^u^**]-RT1.A^a^, but differs in the substituted immunogenic RT1.A^u^ epitope. Comparison of the TCR repertoire in tolerant hosts following allochimeric treatment with either molecule may identify shared CDR3 expansions indicative of regulatory cell clonality induced by shared self-sequences. Our CDR3 spectrotyping analysis of the TCR Vβ genes in allochimeric tolerant rats demonstrated predominant and consistent clonal expansion of the Vβ7, 11, 13, 14 and 15 genes. However, our data showed that the alteration Vβs in allochimeric protein-treated tolerant rats would not occur until after 30 days post-transplantation. This intriguing finding may indicate the establishment of a relatively delayed tolerant state. This phenomenon may be explained by a sequential activation of T cells over time with subsequent spreading of the response [37].

Though it has been demonstrated that maintenance of regulatory cells is dependant on the continuous supply of antigenic stimulation by the allograft [45], it has not been possible to differentiate their potential functional diversity *in vivo* because in most studies, tolerance induction and allograft survival were the endpoints of the analysis [46]. In our studies, we utilized two primary therapies- CsA and allochimeric molecules to achieve distinctive tolerant states, which in turn elicited different responses of TCR repertoire. Such unique TCR restriction model will allow us to examine the functional characteristics and allospecific specificities of regulatory cells based on the inducing therapeutic agent.

## Materials and Methods

### Animals

Adult male inbred Wistar Furth (WF; RT1^u^) and ACI (RT1^a^) rats (200–250 g) were purchased from Harlan Sprague Dawely (Indianapolis, IN) and housed in wire-bottomed cages with controlled light/dark cycle. Rats were given free access to water and standard rat chow and were cared for according to the guidelines of the American Association of Laboratory Animal Care. All experiments were performed according to the NIH standards as set forth in the “Guide for the Care and Use of Laboratory Animals” (DHHS publication No. (NIH) 85–23 Revised 1985). The Institution also accepts as mandatory the PHS “Policy on Humane Care and Use of Laboratory Animals” and NIH “Principles for the Utilization and Care of Vertebrate Animals Used in Testing, Research and Training.

### Site-Directed Mutagenesis and Production of Mutant Class I MHC Molecules

Mutagenesis primers (purified by PAGE) were obtained from *Invitrogen* (Carlsbad, CA). The “QuickChange Multi Site-Directed Mutagenesis Kit” (*Stratagene*, San Diego, CA) was used to mutate ACI-RT1^a^ cDNA as described previously [20], [26] and resulting plasmids were sequenced to confirm the presence of the mutations.

### Production of Allochimeric Protein Containing Mutant Class I MHC Molecules and Allograft Model

The allochimeric proteins containing [α_1h_^u^]-RT1.A^a^ sequence were expressed in transfected Buffalo hepatoma cells as described previously [26]. For tolerance induction, allochimeric proteins were administered through the portal vein (1 mg/rat) into ACI recipients of WF hearts at the time of transplantation followed by a 3-day course of oral cyclosporine delivered by gavage feed (CsA, 10 mg/kg/day; day 0–2). Controls included transplantation of allogeneic hearts in the presence or absence of the same dose of CsA. Heterotopic cardiac transplants were placed intra-abdominally [27].

### TCR Vβ RT-PCR

RNA was extracted from splenocytes and allografted cardiac hearts at post-transplantation day 5, 7, 10, 30, 84, 120 and >120 using the Qiagen RNeasy Mini Kit (QIAGEN, Valencia, CA). In order to remove DNA contamination the RNA samples were digested with DNase I (RNase-free DNase Set, Cat. 79254, QIAGEN) and 25 µg of RNA was reverse transcribed into cDNA (50 µl) by 500 U of SuperScript III reverse transcriptase and SuperScript First-Strand Synthesis System from RT-PCR kit (*Invitrogen*, Carlsbad, CA). Rat Vβ-specific primers were chosen according to Shirwan et al. [28] with minor modifications [29]. A total of 2 µl of cDNA was amplified using a fixed down-stream primer (Cβ1) derived from TCR β chain constant region, and one of the 22 different Vβ-specific upstream primer. Amplification reactions were conducted in a GeneAmp PCR System 9700. The reactions started with a 10 min denaturation step at 94°C, followed by 35 cycles of 30 sec at 94°C, 30 sec at 55°C, and 1 min at 72°C, and ended with an elongation step of 7 min at 72°C.

### Spectrotyping of TCR Vβ CDR3

A 6-FAM-labeled internal primer within the V*β* PCR products (Cβ2 derived from β chain constant region) was synthesized by *Applied Biosystems* (Foster, CA). 7.5 µl of each Vβ-specific PCR product was subjected to 15 cycles of run-off reactions in a final volume of 10 µl, consisting of 0.25 rM of fluorochrome (FAM)-labeled internal primer C*β*2, 0.2 mM of each dNTP, 2 mM MgCl_2_, and 0.2 U AmpliTaq Gold (Perkin Elmer, Foster City, CA) in 1× PCR Gold buffer. Amplification was conducted in a GeneAmp PCR System 9700 (Perkin Elmer), with cycle conditions as follows: denaturation at 95°C for 10 min, 15 cycles of 30 sec at 95°C, 30 sec at 58°C, and 30 sec at 72°C, and elongation at 72°C for 5 min. At the end of the reaction, 5 µl of each spectrotyping reaction was mixed with 10 µl of GeneScan LIZ 500 Size Standard (*Applied Biosystems*, Foster, CA) in 96-well plate. After the denaturation at 94°C for 5 min followed by incubation on ice for 5 min, the 96-well plate was analyzed using 3700 DNA Analyzer (*Applied Biosystems*, Foster, CA).

### Processing of Gel Images

The initial images from DNA Analyzer system were converted into profile plots and the Vβ CDR3 region profiles were visualized using ABI Prism Genotyper 3.7 software. The lanes of each Vβ gene product were selected for profile plot. The plots were then copied to Microsoft Powerpoint files. Two-sample t-test was applied to detect the differences of restriction frequencies of TCR repertoires in CsA and allochimeric protein treated rat tolerant models. Pearson correlation coefficient analysis was performed to determine whether the expression frequency of TCR repertoire correlated with its restriction frequency in allochimeric molecules-induced tolerant recipients. A P value of <0.05 was used to indicate significance.
